# Dissection of Antibody Specificities Induced by Yellow Fever Vaccination

**DOI:** 10.1371/journal.ppat.1003458

**Published:** 2013-06-20

**Authors:** Oksana Vratskikh, Karin Stiasny, Jürgen Zlatkovic, Georgios Tsouchnikas, Johanna Jarmer, Urs Karrer, Michael Roggendorf, Hedwig Roggendorf, Regina Allwinn, Franz X. Heinz

**Affiliations:** 1 Department of Virology, Medical University of Vienna, Vienna, Austria; 2 Division of Infectious Diseases, University Hospital of Zurich, Zurich, Switzerland; 3 Institute for Virology, University of Duisburg-Essen, Essen, Germany; 4 Institute for Medical Virology, University Hospital Frankfurt am Main, Frankfurt am Main, Germany; NIH, United States of America

## Abstract

The live attenuated yellow fever (YF) vaccine has an excellent record of efficacy and one dose provides long-lasting immunity, which in many cases may last a lifetime. Vaccination stimulates strong innate and adaptive immune responses, and neutralizing antibodies are considered to be the major effectors that correlate with protection from disease. Similar to other flaviviruses, such antibodies are primarily induced by the viral envelope protein E, which consists of three distinct domains (DI, II, and III) and is presented at the surface of mature flavivirions in an icosahedral arrangement. In general, the dominance and individual variation of antibodies to different domains of viral surface proteins and their impact on neutralizing activity are aspects of humoral immunity that are not well understood. To gain insight into these phenomena, we established a platform of immunoassays using recombinant proteins and protein domains that allowed us to dissect and quantify fine specificities of the polyclonal antibody response after YF vaccination in a panel of 51 vaccinees as well as determine their contribution to virus neutralization by serum depletion analyses. Our data revealed a high degree of individual variation in antibody specificities present in post-vaccination sera and differences in the contribution of different antibody subsets to virus neutralization. Irrespective of individual variation, a substantial proportion of neutralizing activity appeared to be due to antibodies directed to complex quaternary epitopes displayed on the virion surface only but not on monomeric E. On the other hand, DIII-specific antibodies (presumed to have the highest neutralizing activity) as well as broadly flavivirus cross-reactive antibodies were absent or present at very low titers. These data provide new information on the fine specificity as well as variability of antibody responses after YF vaccination that are consistent with a strong influence of individual-specific factors on immunodominance in humoral immune responses.

## Author Summary

The live-attenuated yellow fever vaccine has been administered to more than 600 million people worldwide and is considered to be one of the most successful viral vaccines ever produced. Following injection, the apathogenic vaccine virus replicates in the vaccinee and induces antibodies that mediate virus neutralization and subsequent protection from disease. In principle, many different antibodies are induced by viral antigens, but it is becoming increasingly clear that only a subset of them is capable of inactivating the virus, and some antibody populations appear to dominate the immune response. However, to date there has been very little information on individual-specific variations of immunodominance and how such variations can affect the functionality of antibody responses. In our study, we addressed these issues and analyzed the fine specificities of antibodies induced by YF vaccination as well as the contribution of different antibody subsets to virus neutralization in 51 vaccinees. We demonstrate an extensive degree of individual variation with respect to immunodominance of antibody populations and their contribution to virus neutralization. Such variations can have an impact on vaccine-mediated protection, and thus insight into this phenomenon can provide leads for novel strategies in modern vaccine design.

## Introduction

The live-attenuated yellow fever (YF) vaccine based on the 17D virus strain is considered to be one of the most successful vaccines ever produced [1], [2]. Since its development in the 1930s by Max Theiler, several hundred million doses have been administered and its effectiveness in protecting from disease has been reported to be at least 90% [1]. Recent studies, including systems biology approaches [3], analyzing innate, cellular and humoral immune responses after YF vaccination indicate that all arms of the immune system are activated, leading to a polyfunctional response that is most likely essential for the long-lasting immunity induced by this vaccine [3], [4], [5]. Despite the broad immunological stimulation, there is strong evidence that humoral immunity mediated by virus-neutralizing antibodies is the primary effector mechanism of protection [1]. Such antibodies may persist for more than 45 years and apparently protect against all naturally occurring genotypes of YF virus [1].

**Figure 1:**
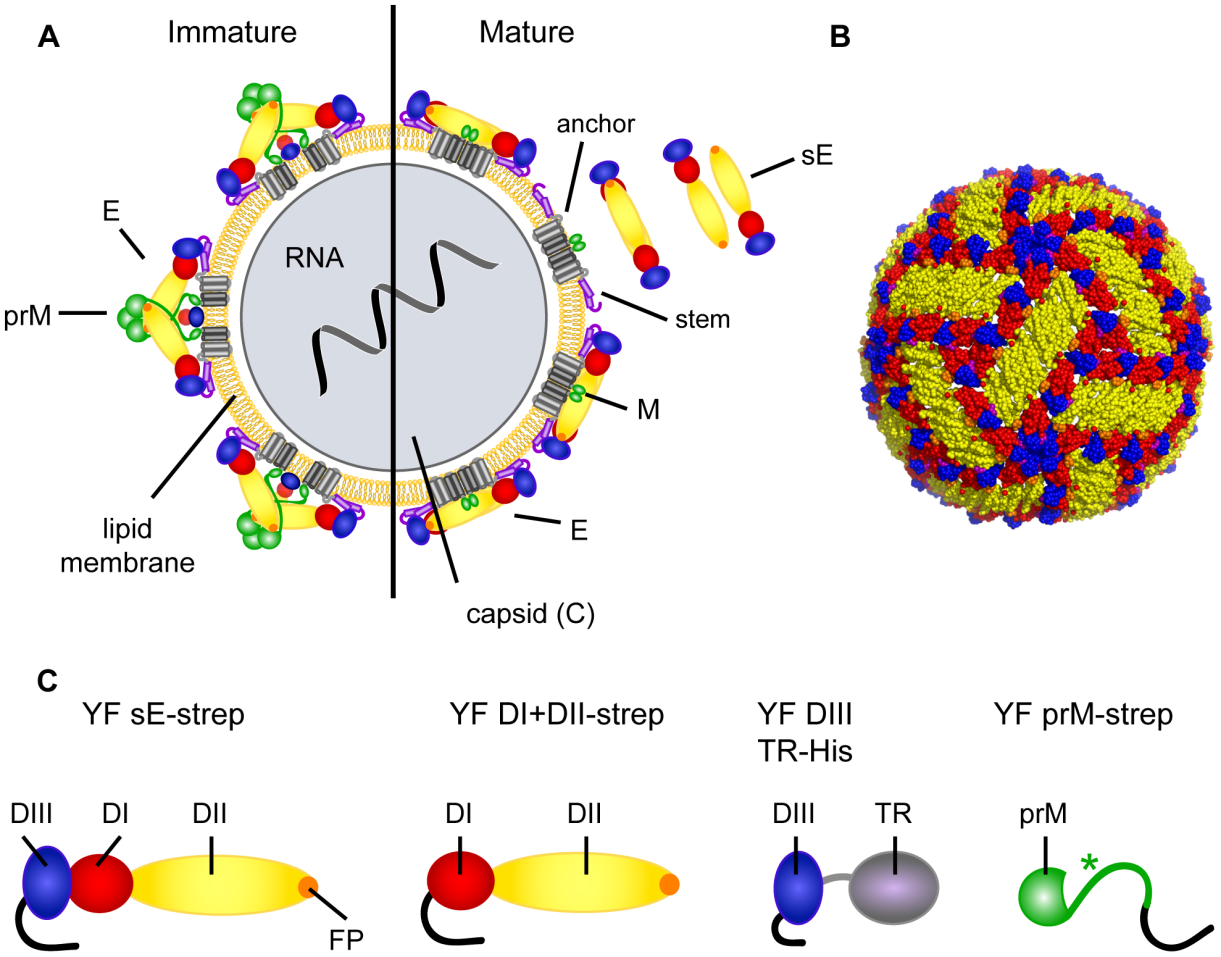
Schematic representation of flavivirus particles and YF virus antigens used in this study. (A) Schematic model of a flavivirus particle. Left panel: immature virion, right panel: mature virion. The capsid contains the viral RNA and multiple copies of the C protein. The surface of immature particles consists of 60 spikes composed of trimers of prM-E heterodimers. Mature particles are formed after prM cleavage and contain 90 E homodimers. The structure of E was determined for soluble forms of E (sE) - schematically shown in the panel of mature virions - lacking the so-called stem- and membrane anchor-regions. (B) Representation of the arrangement of E dimers at the surface of mature dengue 2 virions. Thirty rafts of three parallel E dimers form a herringbone-like icosahedral shell. The figure was constructed using the coordinates of dengue 2 virus from the VIPERdb Virus Particle Explorer (viperdb 1thd; http://viperdb.scripps.edu). (C) Schematic representations of recombinant yellow fever antigens. Color code E: domain I – red, domain II – yellow, domain III – blue; fusion peptide (FP) - orange; stem – purple; transmembrane anchor – light grey. Color code prM: ectodomain – green, transmembrane anchors – dark grey. Color code of recombinant protein tags: black. TR: thioredoxin. The asterisk indicates the position of the mutation in the furin cleavage site of YF prM.

YF virus is the prototypic and name-giving member of the genus flavivirus, family flaviviridae [6]. It is closely related to other mosquito-borne and tick-borne human pathogens, the most important of which are dengue, Japanese encephalitis, West Nile and tick-borne encephalitis viruses [6]. Structural details of flaviviruses have been elucidated for dengue, tick-borne encephalitis (TBE), West Nile (WN), and Japanese encephalitis viruses using X-ray crystallography and cryo-electron microscopy [7], [8], but no such data are yet available for YF virus. However, based on the similarity of the structures determined for different flaviviruses and their close molecular biological and even antigenic relationships, it is justifiable to postulate that the structural organization of YF virus particles as well as its constituting proteins follows the same principles that are typical of flaviviruses in general (Figure 1). Immature virions (Figure 1A, left panel) are assembled in the ER and contain three structural proteins, designated as C (capsid), prM (precursor of membrane) and E (envelope). These particles contain 60 trimeric spikes of prM-E heterodimers and are secreted through the exocytotic pathway of the cell [9]. In the acidic environment of the trans-Golgi network, prM is cleaved by furin [10] and during this process the E protein is completely rearranged to form a herringbone-like lattice of 90 E homodimers at the surface of mature virions [11], [12] (Figure 1A, right panel and Figure 1B). Because of its functions in virus entry (cell attachment and viral fusion in the endosome; [7], [13]), the E protein is the major inducer and target of virus neutralizing antibodies. Neutralizing activity (albeit low) has also been reported for antibodies against prM [14], [15], which is consistent with the fact that the maturation cleavage of prM is not always complete and that partially mature (but already infectious) particles play a role in flavivirus infections [16]. The overall structure of E is highly conserved among all flaviviruses and consists of three characteristically folded domains (DI, DII, and DIII), which are schematically depicted in Figure 1. Studies with other flaviviruses and monoclonal antibodies have shown that antibodies to each of the domains can lead to virus neutralization, although those directed to DIII appeared to have the greatest specific neutralizing activity [17]. The tip of DII contains a highly conserved sequence - the fusion peptide (FP) loop - that is essential in membrane fusion and contributes to the induction of broadly flavivirus cross-reactive antibodies with no or relatively low neutralizing activity [18], [19], [20].

Factors controlling the dominance of antibody responses to different sites on protein antigens are ill-defined but can strongly influence the breadth and functional activity of polyclonal immune sera [21], [22]. Studies on the molecular antigenic structure of flaviviruses have shown that E protein domain-specific, domain overlapping, subunit overlapping, and complex quaternary epitopes can be targets of neutralizing antibody responses, both in animals and humans (reviewed in [8]). Very little information, however, exists with respect to the relative proportions of antibody subsets and individual variations in polyclonal sera after infection or vaccination. The mechanism of B cell stimulation cannot distinguish between sites involved in virus neutralization and ‘decoy’ sites that induce only ‘junk antibodies’ [23], [24], [25], and such variations can have a strong impact on the functional activity of the humoral immune response. Thus, the primary objective of this study was to dissect the polyclonal antibody response after YF vaccination in a large panel of vaccinees and to obtain information on the extent of individual variation as well as its possible consequences for virus neutralization. For this purpose, we made use of the modular organization of the flavivirus E protein and established a platform of immunoassays on the basis of recombinant E protein and its domain substructures (DIII and DI+II) (Figure 1C). We provide evidence that the antibody response to YF vaccination is subject to strong individual variation with respect to the relative proportions of antibodies produced to the domains of E as well as to prM. Importantly, this vaccine appears to induce very little flavivirus cross-reactive antibodies that have been previously shown to dominate immune responses to other flavivirus infections [15], [26], [27], [28]. Antibody depletion experiments strongly suggest that a substantial proportion of the neutralizing antibody response is directed to complex antigenic sites that are not represented by the isolated E protein, but rather are dependent on oligomeric interactions between these proteins at the virion surface.

## Results

### Characteristics of YF vaccinees and sera

**Figure 2:**
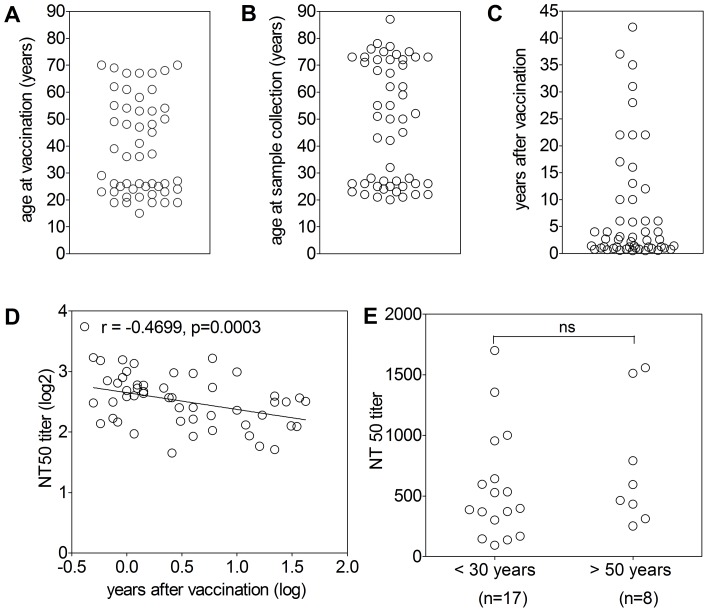
Characteristics of the YF post-vaccination cohort and sera. Distribution of (A) age at the time of vaccination, (B) age at the time of sample collection, and (C) years between vaccination and sample collection. (D) Correlation of YF NT titers and years between vaccination and sample collection. The Pearson correlation coefficients r and the P values are indicated. (E) Comparison of YF NT titers of young (<30 years of age at the time of vaccination) and elderly (>50 years of age at the time of vaccination) vaccinees. Only individuals who were vaccinated within 3 years before sample collection were included. The statistical analysis is provided at the top of the panel (t-test; ns, not significant).

In this study, we analyzed serum samples from volunteers who had been vaccinated with the live YF vaccine in Switzerland and Germany between 0.5 and 42 years prior to sample collection. Since another flavivirus (TBE virus) is endemic in both countries and many people have been vaccinated against this antigenically related virus, we made sure that none of YF vaccinees enrolled in the study had a history of TBE vaccination by interrogation and - as an additional control – by TBE neutralization tests (NT). There was only one serum with a positive TBE NT titer for which we were not able to identify his/her TBE vaccination history and which was excluded from our analyses. The final cohort consisted of 51 individuals which tested positive in YF-NT (titer ≥20) and negative in TBE-NT (titer <10) and had no history of TBE vaccination. The basic characteristics of this cohort with respect to age at vaccination, age at sample collection and time elapsed since vaccination are summarized in Table 1 and displayed in Figure 2A–C. These parameters were not homogeneously distributed, reflecting the age-related travel behavior of YF vaccinees of central Europe. As shown in Figure 2D, a significant negative correlation was found between NT50 titers and the interval of time since vaccination. No statistically significant influence, however, was found with respect to the age at vaccination by comparing the <30 and >50 years old vaccinees that had received the vaccine within 3 years before sample collection (Figure 2E).

**Table 1:** Characterization of cohort of YF vaccinees.

**Number**	51
**Gender**	27m/24f
**Age at YF vaccination**	36* (15–70)**
**Age at sample collection**	50* (20–87)**
**Years after YF vaccination**	2.58* (0.5–42)**
**TBE NT**	Negative (NT titer <10)

* median;

** range.

### Quantification of antibody subsets in polyclonal sera and their relative proportions

For dissecting the specificities of antibody populations present in the 51 post-vaccination sera and the contribution of such antibody subsets to virus neutralization, we produced the following set of C-terminally strep- or His-tagged recombinant YF proteins as shown in Figure 1C: 1. a C-terminally truncated soluble monomeric form of YF E (YF sE-strep); 2. domains DI+II of YF E (YF DI+II-strep); 3. domain III of YF E as a fusion protein with thioredoxin (YF DIII TR-His); and 4. a C-terminally truncated soluble form of YF prM (YF prM-strep). To analyze the presence of flavivirus cross-reactive antibodies, we used sE proteins from TBE virus (TBE sE-strep) and WN virus (WN sE). Evidence for proper folding was obtained in a number of control experiments, which are described in detail in the supporting Information (Figure S1, Text S1 and S2).

**Figure 3:**
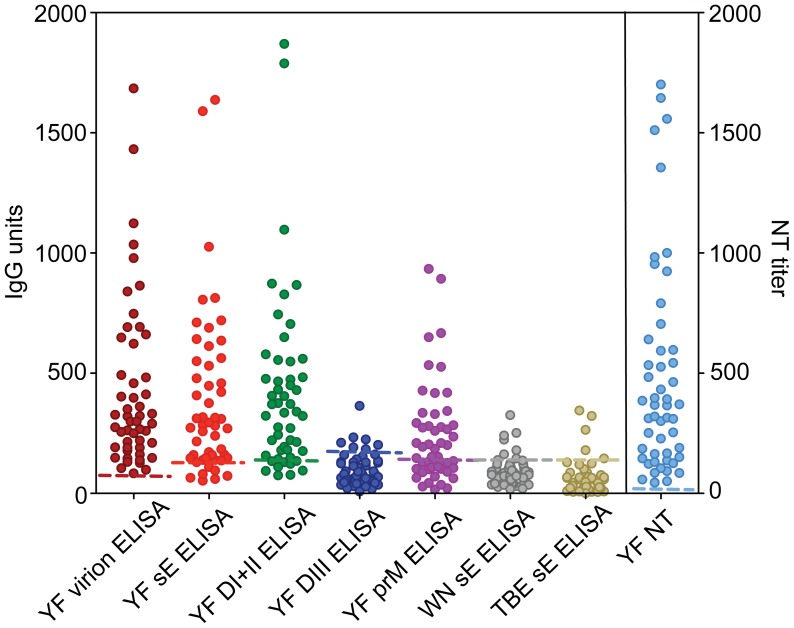
ELISA reactivities and NT titers of YF post-vaccination sera. Antibody ELISA reactivities against different antigens are expressed as IgG units. Dashed lines indicate the cut-off in each assay.

Using these recombinant proteins, we established ELISAs that were all highly sensitive and specific (see Supplemental materials, Figure S2AB, Text S1 and S2) and – with the use of internal standards - allowed us to quantify antibodies directed to E and its substructures as well as to prM in relation to those measured in virion ELISAs and virus neutralization assays (Figure S3, Text S1 and S2). The results obtained in these assays with the YF post-vaccination sera from the 51 individuals are shown in Figure 3. All of the sera were positive in the virion ELISA and neutralization assay (with complete neutralization at low serum dilutions in all instances), compared to 82% positives in the sE and DI+II ELISAs, respectively. Remarkably, only 14% low positives were observed in the ELISAs using YF DIII or sE of distantly related flaviviruses (WN and TBE viruses), although the corresponding assays had sensitivities similar to those of the YF sE and DI+II ELISAs (see Figure S2A for YF DIII ELISA and Figure S2BC for WN and TBE ELISAs). Antibodies to prM were somewhat intermediate, with 57% of the post-vaccination sera being positive. Statistical analyses showed highly significant positive correlations (p<0.0001) between all of the units/titers measured in the different antibody assays (Table 2).

**Table 2:** Correlation of results obtained in different YF antibody assays (Pearson correlation coefficients).

Assay	sE ELISA	DI+II ELISA	prM ELISA	virion ELISA
sE ELISA				
DI+II ELISA	0.96 (p<0.0001)			
prM ELISA	0.84 (p<0.0001)	0.88 (p<0.0001)		
virion ELISA	0.89 (p<0.0001)	0.93 (p<0.0001)	0.90 (p<0.0001)	
NT	0.58 (p<0.0001)	0.63 (p<0.0001)	0.59 (p<0.0001)	0.65 (p<0.0001)

**Figure 4:**
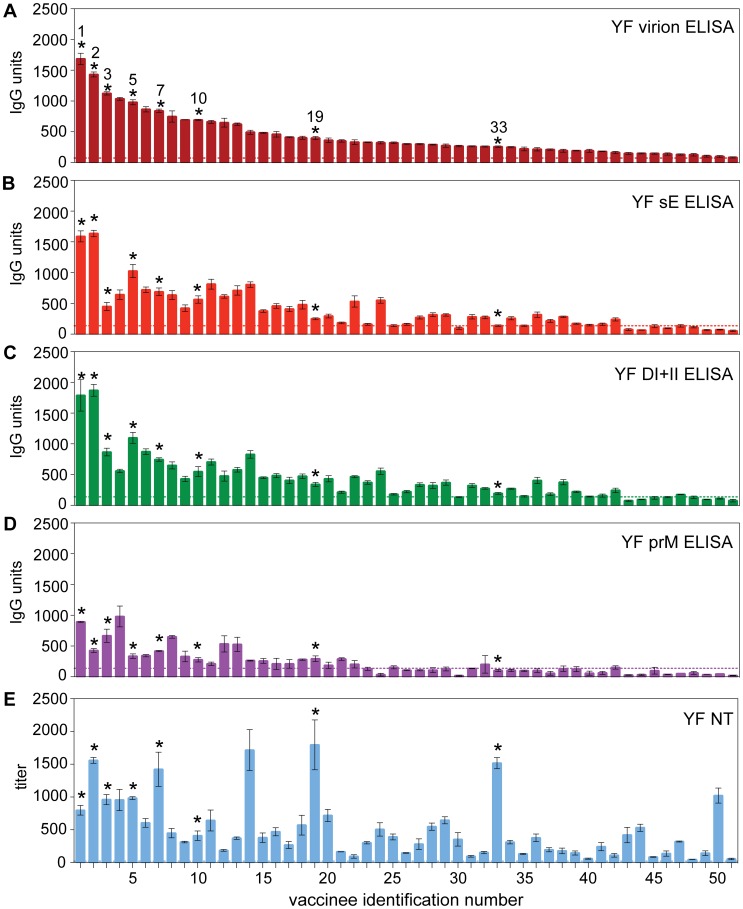
ELISA reactivities and NT titers of YF post-vaccination sera ordered according to their virion ELISA reactivities. ELISA IgG units obtained with YF virion (A), YF sE (B), YF DI+II (C) and prM (D). (E) YF virus-specific NT titers. Dashed lines indicate the cut-off in each assay. Numbers and asterisks on top of the columns indicate sera of vaccinees selected for depletion analyses (Table 3 and Figure 6).

**Figure 5:**
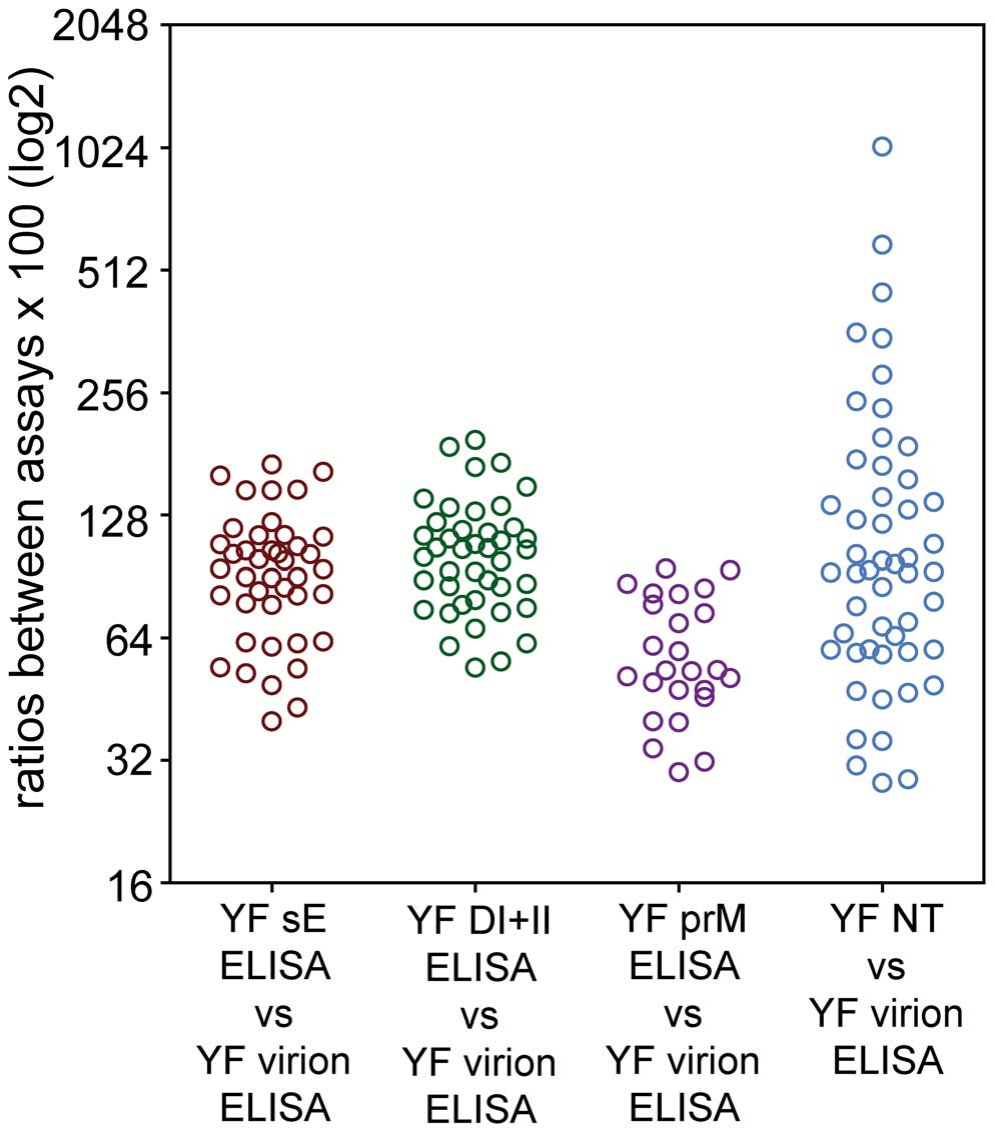
Extent of variation of antibody reactivities in YF post-vaccination sera. The ratio of results obtained in the YF sE, YF DI+DII and YF prM ELISA as well as YF NT relative to virion ELISA results was calculated for all sera. Data are expressed as log2 of these ratios multiplied by 100 and each of the circles represents the ratio obtained for an individual serum.

Independent of the differences observed with respect to antibody titers (at least in part due to the different time windows between vaccination and blood sampling; Figure 2D), our primary interest in this study was directed at individual-specific variations in the composition of antibody specificities and their proportions in post-vaccination sera as well as their possible influence on virus neutralization. To identify such variations, we plotted the reactivities in the virion ELISA (Figure 4A) against those of the sE, DI+II and prM ELISAs (Figure 4B–D) as well as NTs (Figure 4E). DIII-reactivities were not included because they were mostly negative. This analysis revealed substantial deviations from the order of reactivities in the virion ELISA, providing evidence for different antibody compositions of individual sera that also affected their functional activities in virus neutralization. To quantify the extent of this variation, we calculated the ratios of antibody units obtained in sE, DI+II, and prM-ELISAs as well as NT titers relative to virion ELISA units for each individual serum sample. These ratios are shown in Figure 5, which reveals a substantial degree of variation, especially in the case of the NT/virion ELISA ratios. In the subunit ELISAs, sera yielding negative results were omitted from these calculations and the real extent of variation is most likely higher than displayed in the figure. In other viral systems, the possibility of a selective decline of certain antibody specificities has been reported. Since the time between vaccination and sample collection varied substantially between individuals and differences in the decline of antibody subsets cannot be excluded *a priori*, we assessed the correlation between the ratios shown in Figure 5 and the time elapsed since vaccination. The Pearson correlation coefficients between these parameters, however, were 0.07 to −0.17 (p values≥0.3), showing that there was no significant change in antibody composition of post-vaccination sera over time.

### Depletion analysis

**Figure 6:**
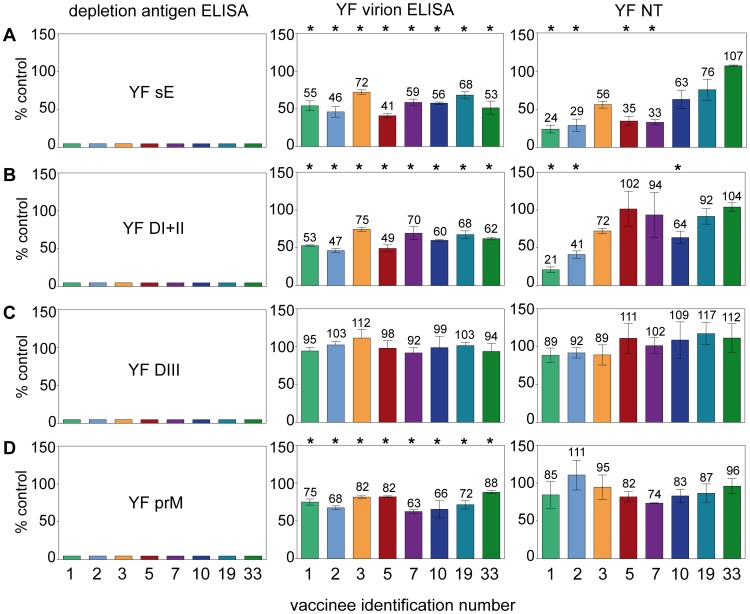
ELISA and NT analyses of YF post-immunization sera after depletion with YF sE (panels A), YF DI+II (panels B), YF DIII (panels C) and YF prM (panels D). Left panels: Percent reactivity in post-depletion sera, determined by ELISA with the depletion antigen. Middle panels: Percent reactivity in post-depletion sera, determined by ELISA with the virion. Right panels: Percent reactivity in post-depletion sera, determined by NT. Results are expressed as percent of the ELISA IgG units or NT titers of mock-depleted sera (control). The numbers above the columns in the middle and right panels indicate the percentage of reactivities remaining after depletion. Asterisks indicate the significance of difference in antibody reactivities between depleted and control sera (t-test). The error bars represent standard error of the means calculated from the results of at least three independent assays. Identification numbers of vaccinees are indicated under the panels (compare with Table 3 and Figure 4).

To obtain information on the contribution of different antibody populations to virus neutralization in individual sera, we conducted depletion analyses by removing distinct antibody subsets with the recombinant YF antigens sE, DI+II, DIII, and prM bound to magnetic beads. The complete removal of antibodies was controlled by performing an ELISA with the antigen used for depletion (Figure 6, left panels), and then the residual reactivity of the depleted sera was analyzed in the virion ELISA (Figure 6, middle panels) and NT (Figure 6, right panels) compared to sera mock-depleted with magnetic beads only as the 100% control. A set of 8 serum samples was selected for these analyses, which all had relatively high NT titers and differed in their ratios of reactivities with the recombinant antigens. As an example, the original data of the depletion analyses for vaccinee 2 are provided in Figure S4 which shows that the curves before and after depletion were almost parallel. The characteristics of all 8 vaccinees (marked with asterisks in Figure 4) are summarized in Table 3 and the results of depletion analyses are presented in Figure 6. Depletion with DIII (Figure 6, panels C) did not result in any significant reduction of the virion ELISA- and NT-reactivities, consistent with the low or negative titers measured in DIII ELISA (compare Figure 3). In contrast, substantial proportions of antibodies were removed by sE and DI+II from many (but not all) serum samples, ranging from 25 to 59% of the virion ELISA reactivity and 0 to 79% of the NT-activity (Figure 6, panels A and B). In some instances, there was a good correlation between the depletion measured in the virion ELISA and NT, and in other cases substantial deviations were observed, which are specifically addressed below. The depletion assays also confirmed the induction of prM-specific antibodies by YF vaccination (ranging from 12 to 37% of the total virion ELISA reactivity) but their contribution to virus neutralization (ranging from 0 to 26%) was not statistically significant (Figure 6D, right panel; p>0.05). Overall, this analysis confirmed a high degree of individual variation in the composition of post-vaccination sera with respect to virion-specific and neutralizing antibodies.

**Table 3:** Characteristics of sera selected for depletion.

					Assay ratios * ×100			
Vaccinee (nr.)	Age at vaccination (years)	Age at sample collection (years)	Years after vaccination	Gender	NT vs virion ELISA	sE ELISA vs virion ELISA	DI+II ELISA vs virion ELISA	prM ELISA vs virion ELISA
1	61	62	0.92	m	47	94	106	53
2	58	59	0.92	f	109	114	130	30
3	19	22	2.67	f	85	40	77	59
5	41	51	10	m	10	10	11	3
7	26	28	1.17	f	16	8	9	5
10	27	28	1.17	m	57	81	79	40
19	26	26	0.5	f	427	78	85	74
33	67	67	0.57	m	59	5	8	4

* Ratios of YF neutralizing antibodies as well as YF sE, YF DI+DII and YF prM ELISA IgG units versus YF virion ELISA IgG units.

Because of their distinctive patterns, the results obtained with the sera of vaccinees 1 and 33 on the one hand and vaccinees 5 and 7 on the other are especially noteworthy. In the case of serum from vaccine 33, almost half of the virion reactivity was removed by depletion with sE (as well as DI+II); however, in contrast to other sera this did not result in any significant reduction in neutralizing activity. Since DIII-specific and prM-specific antibodies also did not contribute to neutralization in this case, we conclude that antibodies to subunit-overlapping (i.e., dimer-specific) and/or herringbone-specific quaternary epitopes (but not antibodies to the monomeric sE) were primarily responsible for the neutralizing activity of this serum. The opposite was seen with serum from vaccinee 1. Similar to serum from vaccinee 33, approximately 50% of the virion reactivity was removed by sE and DI+II, but in contrast, the neutralization effect was substantial and accounted for more than 75% of the total neutralizing activity (Figure 6A, right panel).

The similarity and excellent correlation of reactivities observed in sE and DI+II ELISAs (Figure 4 and Table 2) was also confirmed when sE- and DI+II-depleted sera were analyzed in the virion ELISA (Figure 6A and B, middle panels). However, a significant discrepancy was observed for NT of sera from vaccinees 5 and 7. In both cases, approximately 65% of the total neutralizing activity was depleted by sE, whereas DI+II had no measurable effect. Since there was no evidence for DIII antibodies in these sera (Figure 6C), it is likely that antibodies to epitopes at the junction between DI and DIII (present in sE but not in DI+II) were responsible for these results. From these data, we conclude that individual vaccinees not only differ dramatically with respect to the composition of their antibody subsets directed to different parts of viral surface proteins, but also with respect to the contribution of such subsets to virus neutralization.

## Discussion

The primary goal of this study was to gain insight into individual variations in fine specificities of the antibodies and their possible impact on virus neutralization after YF vaccination. This was accomplished by quantifying subsets of antibodies directed to distinct domains of the viral envelope protein E and determining their contribution to virus neutralization. Our data provide evidence for extensive differences in the specificities and relative proportions of antibody populations induced by YF vaccination in different individuals. This conclusion is primarily based on the observation that the ratios of reactivities in ELISAs with the monomeric E, DI+II and prM relative to the virion ELISA reactivities varied substantially (compare Figure 5). Substantial variation was found in the ratio between virion ELISA reactivities and neutralization titers, suggesting a strong influence of antibody subset composition on the functional activity of individual sera. Further confirmation of the observed heterogeneities was obtained by the quantitative analysis of antibodies in sera depleted with recombinant antigens. Specifically, depletion with the monomeric sE resulted in a strongly diverging pattern of neutralizing activity removed (ranging from 0 to 79%) which in several instances did not match the reactivity pattern of the virion ELISA (compare the panels in Figure 6 A), and similar differences were also observed in depletions with DI+II. Our data not only demonstrate extensive differences in the fine specificities of antibody subsets in post-vaccination sera, but also that these heterogeneities can strongly affect functional activities such as virus neutralization. It is likely that these findings are related to cooperative and/or competitive interactions between antibody populations directed to the same target antigen but displaying different fine specificities, avidities and concentrations. Such effects have been described in studies with monoclonal antibodies [29], [30], [31] and were proposed for explaining variations in the efficiency of polyclonal sera to neutralize influenza virus [32] and HIV [31]. In the case of YF and other flaviviruses, the phenomenon of virus breathing has to be considered as an additional layer of complexity, because it allows binding of antibodies to epitopes that are seemingly inaccessible in a static model of virion structure but become exposed through dynamic motions of the virion shell [33], [34], [35], [36], [37], [38]. Antibodies to such sites can thus contribute to virus neutralization and increase the potential individual variation.

Studies with dengue viruses as well as other flaviviruses have shown that genotype- or strain-variations affecting individual epitopes and/or the degree of virus maturation can have a profound effect on the results of neutralization assays [14], [39], [40], [41], [42]. Since the infecting strain is rarely known in studies with post-infection sera, neutralization results generated with a specific laboratory strain may be biased by such phenomena. In this context, our study had the advantage of using antigens in the ELISAs as well as the virus in neutralization assays that were identical to the 17D virus strain used for immunization. The variations observed can therefore be considered true variations in fine specificities of antibodies for the same immunogen in different individuals, which are not influenced by possible strain-specific effects.

One important finding of our study is that a varying proportion of neutralizing antibodies induced by YF vaccination in individuals is apparently directed to complex epitopes found at the virion surface only, but not on an isolated form of the monomeric E protein. In all instances, only part of the total neutralizing activity could be removed by sE depletion, suggesting that the residual activity is due to antibodies specific for E dimer-dependent or even more complex dimer-overlapping epitopes that are generated by the herringbone-like quaternary arrangement of E dimers at the virion surface (Figure 1B). Indeed, an epitope comprising residue 71 in DII and 155 in DI of the opposing E monomer was identified as a dimer-specific epitope by the use of human monoclonal antibody fragments derived by repertoire cloning from YF patients [43]. E-dimer- and herringbone-dependent epitopes have also been characterized for other flaviviruses using monoclonal antibodies [44], [45], [46], [47] and - in the case of post-infection dengue sera - the majority of antibodies seemed to be directed against such complex epitopes [48]. In some YF post-vaccination sera, substantially different patterns of neutralizing activity were also observed after depletion with DI+II compared to sE (Figure 6AB, right panels). Since antibodies to DIII did not contribute to the depletion results in these cases, it can be assumed that the discrepancies observed were due to antibodies directed to epitopes at the junction between DI and DIII.

In contrast to the high frequencies and titers of antibodies against DI+II, only a very low proportion of the post-vaccination sera had antibodies against DIII (only 14% were positive) and the titers of positive sera were low compared to those against sE and DI+II, although the assays had comparable sensitivities (Figure S2A). Furthermore, depletion analyses did not reveal any contribution of DIII antibodies to virus neutralization in a set of 8 selected serum samples (Figure 6). DIII-specific antibodies had been shown to dominate humoral immune responses in the mouse [27], [49] and to exhibit a higher specific neutralizing activity than antibodies to other sites in E [17]. As revealed in several recent studies, however, DIII-specific antibodies appear to play only a minor role in human antibody responses to flaviviruses (reviewed in [8], [50]). Our results are consistent with data on human immune responses to dengue [26], [51], [52] as well as West Nile [27], [45] virus infections, which demonstrate that DIII-responses formed only a very small proportion of the total antibody response in these species and that most of the neutralizing activity was due to antibodies directed to other sites in E [51], [52].

Similar to the situation with DIII-specific antibodies, we also found only very low frequencies and titers of broadly flavivirus cross-reactive antibodies. Such antibodies were shown to recognize epitopes around the highly conserved FP loop (Figure 1) and usually do not contribute significantly to virus neutralization [18], [19], [20]. Our data are consistent with previous reports on highly type-specific antibody responses after primary YF vaccination measured in hemagglutination inhibition assays and also in ELISA [53], [54] but differ from the antibody response to dengue virus infections. In the latter case, FP-specific antibodies were shown to make up a large proportion of the total antibody response [26], [28]. The reasons for these discrepancies are presently unclear but may be related to differences in the formation and/or structure of partially immature particles during virus replication in dengue-infected individuals and YF17D vaccinees. The degree of virus maturation (i.e. cleavage of prM) has been shown to affect FP exposure by the demonstration of enhanced accessibility to antibodies [55] and thus can potentially have a profound effect on post-infection antibody profiles.

In contrast to the low DIII- and FP-specific responses, a substantial proportion (57%) of the YF vaccinees in our study had antibodies to prM. Studies on prM responses after flavivirus infections are still limited but Western blot analyses have detected prM antibodies in dengue and JE post-infection sera [56], and a surprisingly high proportion of human monoclonal antibodies generated from dengue infected individuals were found to be prM-specific and able to promote antibody-dependent enhancement of infection [14], [15], [57]. In our depletion analysis, we found a moderate (but not statistically significant) contribution of prM antibodies to virus neutralization (Figure 6D, right panel), which is consistent with the poor neutralizing activity of dengue virus prM antibodies [14]. It is likely that these neutralization data are strongly influenced by the maturation state of the virus used in the assays [14], [42] and therefore further studies using artificial viruses with defined prM-content will be necessary to compare the extent of prM-responses after different flavivirus infections as well as assess their role in virus neutralization, infection enhancement and protection.

In conclusion, our study demonstrates a high degree of individual variability in the fine specificities of antibody responses to YF vaccination which affects virus neutralization. It is currently unclear, whether and to what extent such variations can impact the protective efficacy of YF vaccination and are related to rare but existing vaccination failures [2]. Our data are consistent with the assumption that the phenomenon of antibody immunodominance is strongly influenced by individual factors that control the selection of high-affinity B cell clones for antibody production, in addition to possible structural factors intrinsic to the antigen. Such individual variations have to be considered in structure-based vaccine designs that attempt to target the immune response to the most potent protective antigenic sites [58].

## Materials and Methods

### Human sera

Serum samples were collected from individuals 0.5 to 42 years after YF vaccination at the Institute of Virology, University of Duisburg-Essen, Germany, the Institute of Medical Virology, University Clinic of Frankfurt, Germany, and the Division of Infectious Diseases and Hospital Epidemiology, University Hospital of Zuerich, Switzerland. In total, 51 serum samples with no records of other flavivirus infections or vaccinations were sent to the Department of Virology, Medical University of Vienna, Austria for diagnostic analyses and were used anonymously in this study.

### Ethics statement

The studies were approved by the ethics committees of the University of Duisburg-Essen, Germany, the University Clinic of Frankfurt, Germany, the University Hospital of Zuerich, Switzerland, and the Medical University of Vienna, Austria.

### Virus production

A suckling mouse brain suspension of the YF virus 17D-204 vaccine strain was used as an inoculum for propagating the virus in Vero cells using Dulbecco's Modified Eagle Medium (DMEM) supplemented with 0.1% bovine serum albumin (BSA). Cell supernatants were harvested 72 h post-infection, clarified by centrifugation at 10,000 g (30 min; 4°C), concentrated by ultracentrifugation at 150,000 g (1 h; 4°C) and purified by rate zonal sucrose gradient ultracentrifugation as described for TBE virus [59]. The virus peak was identified by hemagglutination assays using goose red blood cells at pH 6.4 as described by Clarke and Casals [60].

### Production of recombinant proteins

The sE (aa 1–397), DI-II (aa 1–294) and prM (aa 1–129) proteins of YF virus 17D (GenBank accession number X03700), as well as sE (aa 1–400) of TBE virus strain Neudörfl (GenBank accession number U27495) were produced in Drosophila Schneider 2 (S2) cells using the pT389 vector (provided by Thomas Krey and Felix Rey, Institut Pasteur, France), which encodes the Drosophila export signal sequence BiP, an enterokinase cleavage site and a double strep-tag. All of these proteins were produced in soluble form by C-terminal truncations that removed their membrane anchors. In the case of the YF prM protein, the furin cleavage site was mutated to obtain unprocessed prM, as previously described [61]. WN sE (aa 1–400) was produced in Drosophila Schneider 2 (S2) cells using the pMTBip/V5-His vector (Life Technologies), with a stop-codon introduced after the sE-sequence to yield the expression of the protein without a His-tag as previously described [49]. Transfection of S2 cells was carried out with CaCl_2_ according to the manufacturer's protocol (Invitrogen) and blasticidin resistance was used for the selection of stably transfected cells. Recombinant protein expression was induced by CuSO_4_, and the supernatants were harvested 7 d post-induction. Strep-tagged recombinant proteins were purified using Strep-Tactin columns (IBA), - according to the manufacturer's protocol, and the untagged WN sE protein was purified by immunochromatography using the flavivirus cross-reactive antibody 4G2 (ATCC) as previously described [49].

DIII of YF virus 17D (aa 295–391 in E) was expressed in *E. coli* BL-21 cells as a fusion protein with thioredoxin and a C-terminal His tag using the pET 32a Xa/LIC vector (Novagen). In this vector, the internal His-tag was removed from the expression cassette by site-directed mutagenesis (Life Technologies), leaving only the C-terminal His-tag [49]. The recombinant fusion protein was purified from clarified bacterial cell lysates by Ni^2+^ affinity chromatography (GE Healthcare Life Sciences) following the manufacturer's protocol.

Schematics of the YF recombinant proteins are shown in Figure 1.

### IgG ELISA

ELISAs for analyzing YF virus-specific antibodies were performed essentially as previously described [18]. Briefly, microtiter plates were coated overnight at 4°C with pre-determined optimized dilutions of purified recombinant antigens or virus in carbonate buffer (pH 9.6). Plates were blocked with phosphate-buffered saline (PBS) pH 7.4 containing 2% lamb serum for 20 min at 37°C. Threefold serial dilutions (starting at 1∶100) of human sera were then added for 1 h at 37°C. Biotin-labeled goat anti-human IgG (Pierce) together with Streptavidin–Peroxidase (Sigma) was used for detection. Sera were analyzed in at least three independent experiments and specific IgG was quantified using human YF post-vaccination sera - arbitrarily defined to contain 1000 IgG Units – as internal standards (see Supporting information). Four dilutions of each sample were analyzed and data points within the linear range of the standard curves were used for determining IgG units. The cut-off was determined in each test by including flavivirus-negative human sera and set at the mean plus three standard deviations.

### YF virus neutralization assay

Neutralization assays were carried out in baby hamster kidney cells (ATCC BHK-21) using two-fold serial dilutions of sera (in triplicates) - starting at a dilution of 1∶20 – and the YF vaccine virus propagated in suckling mouse brain. The serum samples were incubated with 20–40 TCID_50_ virus for 1 h at 37°C before the addition of cells, which were then incubated for three additional days. After removal of cell supernatants, the cells were fixed with 4% paraformaldehyde for 20 min at room temperature, and treated with a Tris-buffer (50 mM Tris, 150 mM NaCl, pH 7.6) containing 3% nonfat dry milk, 0.5% Triton X-100, and 0.05% Tween 20 for 30 min at 37°C. The YF virus-specific monoclonal antibody 2D12 (ATCC) was then added and the fixed cells were incubated for 1.5 h at 37°C. Bound antibodies were detected with alkaline phosphatase-labeled anti-mouse IgG (Sigma) and SigmaFast pNNp (Sigma) as a substrate. The enzymatic reaction was stopped with 1.5 N NaOH after 30 min and the absorbance was measured at 405 nm. Titers were determined after curve fitting with a four-parameter logistic regression (GraphPad Prism 5; GraphPad Software Inc.) using 50% of the absorbance in the absence of antibody as a cut-off (NT50). Titers ≥20 were considered positive.

### Antibody depletion assays

Antibody depletion was essentially performed as previously described [49] using the “Dynabeads His-Tag Isolation&Pulldown kit” (Life Technologies) for binding proteins containing a His-tag and Strep-Tactin magnetic beads (Qiagen) for binding proteins containing a strep-tag. Thirty micrograms of recombinant proteins were incubated with 100 µl paramagnetic beads and divided into three aliquots. After pelleting by magnetic force, the beads were resuspended in a buffer according to the manufacturer's instructions and incubated for 1 h at 37°C with a 1∶5 dilution of serum. The beads were pelleted by magnetic force and the depleted serum was collected. To achieve quantitative depletion, this procedure was performed three times. Absence of non-specific binding of the antibodies to the beads was controlled by incubating sera with unloaded beads.

### Statistical analyses

Statistical analyses were conducted using GraphPad Prism 5 (GraphPad Software Inc.). Logarithmic transformations of data were performed to obtain approximate normal distribution of IgG arbitrary units and NT titers. Two-tailed t-tests were used to compare the transformed data and Pearson correlation tests were used to determine correlation coefficients. P values<0.05 were considered statistically significant.

### Supporting information

Supporting information includes four figures (Figures S1, S2, S3, S4) and a description of these figures (Text S1 and S2).

## Supporting Information

Figure S1 Characterization of recombinant antigens. (A) SDS-PAGE of recombinant antigens under reducing (+β-mercaptoethanol) conditions. (B) SDS-PAGE of untreated (−) and DMS cross-linked (+) YF sE-strep and TBE sE-strep proteins. The positions of the sE monomer (M) and dimer (D) are indicated. (C) Sucrose gradient sedimentation analysis of the YF sE-strep (left panel) and TBE sE-strep (right panel) at pH 8.0 and pH 6.0. The sedimentation direction is shown from left to right, and the positions of the sE monomer (M) and dimer (D) are indicated. (D) SDS-PAGE of non-treated (−) and PNGase F-treated (+) YF sE-strep, YF DI+II-strep and YF prM-strep proteins. (E) Western blot of non-reduced (−) and reduced (+) recombinant YF sE-strep, YF DI+II-strep and YF fusion DIII-His using conformation-sensitive monoclonal antibodies as well as non-reduced (−) and reduced (+) YF prM-strep with a polyclonal rabbit serum.
(TIF)

Figure S2 Sensitivities and specificities of YF virion and recombinant antigen ELISAs for the detection of YF virus-specific antibodies. (A) Reactivity with the DIII-specific MAb 86.64. (B) Reactivity with the cross-reactive fusion peptide loop-specific MAb A1. (C) Reactivity with TBE virus post-vaccination sera.
(TIF)

Figure S3 ELISA reactivities of standard sera with YF virion (A), YF sE (B), YF DI+II (C), YF DIII (D), YF prM (E), and WN sE (F). Error bars represent standard deviations, which were calculated from at least three independent experiments.
(TIF)

Figure S4 ELISA and NT analyses of serum from vaccinee 2 (Figure 6) after depletion with YF sE (A), DI+II (B), DIII (C) and prM (D). Left panels: absorbance curves before and after depletion, determined in ELISA with the depletion antigen. Middle panels: absorbance curves before and after depletion, determined in ELISA with the YF virion. Right panels: Neutralization curves before and after depletion. Error bars represent standard errors of the mean, which were calculated from at least three independent experiments. All panels: red curves indicate sera before depletion, grey curves mock depletions, green curves sera after depletion, dashed grey lines cut-offs. IgG ELISA units and NT titers before and after depletion were determined as described in Materials and Methods.
(TIF)

Text S1 Text S1 describes the characterization of recombinant proteins used in the study and the standardization of ELISAs with these antigens.
(DOCX)

Text S2 Text S2 describes Materials and Methods pertaining to data shown in the supporting figures.
(DOCX)

## References

[1] Barrett AD, Teuwen DE (2009) Yellow fever vaccine - how does it work and why do rare cases of serious adverse events take place? Curr Opin Immunol 21: 308–313. 19520559 10.1016/j.coi.2009.05.018

[2] Monath TP, Gershman M, Staples JE, Barrett AD (2012) Yellow fever vaccine. In: Plotkin SA, Orenstein WA, Offit PA, editors. Vaccines: Saunders Elsevier. pp. 870–968.

[3] Querec TD, Akondy RS, Lee EK, Cao W, Nakaya HI, et al (2009) Systems biology approach predicts immunogenicity of the yellow fever vaccine in humans. Nat Immunol 10: 116–125. 19029902 10.1038/ni.1688 PMC4049462

[4] Pulendran B (2009) Learning immunology from the yellow fever vaccine: innate immunity to systems vaccinology. Nat Rev Immunol 9: 741–747. 19763148 10.1038/nri2629

[5] Querec TD, Pulendran B (2007) Understanding the role of innate immunity in the mechanism of action of the live attenuated Yellow Fever Vaccine 17D. Adv Exp Med Biol 590: 43–53. 17191376 10.1007/978-0-387-34814-8_3

[6] Simmonds P, Becher P, Collett MS, Gould EA, Heinz FX, et al.. (2011) Family Flaviviridae. In: King AMQ, Lefkowitz E, Adams MJ, Carstens EB, editors. Virus Taxonomy IXth Report of the International Committee on Taxonomy of Viruses. San Diego: Elsevier Academic Press.

[7] Kaufmann B, Rossmann MG (2011) Molecular mechanisms involved in the early steps of flavivirus cell entry. Microbes Infect 13: 1–9. 20869460 10.1016/j.micinf.2010.09.005 PMC3014442

[8] Heinz FX, Stiasny K (2012) Flaviviruses and their antigenic structure. J Clin Virol 55: 289–295. 22999801 10.1016/j.jcv.2012.08.024

[9] Lindenbach BD, Thiel HJ, Rice CM (2007) Flaviviridae: The viruses and their replication. In: Knipe DM, Howley PM, Griffin DE, Lamb RA, Martin MA et al.., editors. Fields Virology. 5 ed. Philadelphia: Lippincott. Williams & Wilkins. pp. 1101–1152.

[10] Stadler K, Allison SL, Schalich J, Heinz FX (1997) Proteolytic activation of tick-borne encephalitis virus by furin. J Virol 71: 8475–8481. 9343204 10.1128/jvi.71.11.8475-8481.1997 PMC192310

[11] Mukhopadhyay S, Kim BS, Chipman PR, Rossmann MG, Kuhn RJ (2003) Structure of West Nile virus. Science 302: 248. 14551429 10.1126/science.1089316

[12] Kuhn RJ, Zhang W, Rossmann MG, Pletnev SV, Corver J, et al (2002) Structure of dengue virus: implications for flavivirus organization, maturation, and fusion. Cell 108: 717–725. 11893341 10.1016/s0092-8674(02)00660-8 PMC4152842

[13] Stiasny K, Fritz R, Pangerl K, Heinz FX (2011) Molecular mechanisms of flavivirus membrane fusion. Amino Acids 41: 1159–1163. 19882217 10.1007/s00726-009-0370-4

[14] Dejnirattisai W, Jumnainsong A, Onsirisakul N, Fitton P, Vasanawathana S, et al (2010) Cross-reacting antibodies enhance dengue virus infection in humans. Science 328: 745–748. 20448183 10.1126/science.1185181 PMC3837288

[15] Beltramello M, Williams KL, Simmons CP, Macagno A, Simonelli L, et al (2010) The human immune response to Dengue virus is dominated by highly cross-reactive antibodies endowed with neutralizing and enhancing activity. Cell Host Microbe 8: 271–283. 20833378 10.1016/j.chom.2010.08.007 PMC3884547

[16] Pierson TC, Diamond MS (2012) Degrees of maturity: the complex structure and biology of flaviviruses. Curr Opin Virol 2: 168–175. 22445964 10.1016/j.coviro.2012.02.011 PMC3715965

[17] Pierson TC, Fremont DH, Kuhn RJ, Diamond MS (2008) Structural insights into the mechanisms of antibody-mediated neutralization of flavivirus infection: implications for vaccine development. Cell Host Microbe 4: 229–238. 18779049 10.1016/j.chom.2008.08.004 PMC2678546

[18] Stiasny K, Kiermayr S, Holzmann H, Heinz FX (2006) Cryptic properties of a cluster of dominant flavivirus cross-reactive antigenic sites. J Virol 80: 9557–9568. 16973559 10.1128/JVI.00080-06 PMC1617264

[19] Crill WD, Chang GJ (2004) Localization and characterization of flavivirus envelope glycoprotein cross-reactive epitopes. J Virol 78: 13975–13986. 15564505 10.1128/JVI.78.24.13975-13986.2004 PMC533943

[20] Vogt MR, Dowd KA, Engle M, Tesh RB, Johnson S, et al (2011) Poorly neutralizing cross-reactive antibodies against the fusion loop of West Nile virus envelope protein protect in vivo via Fcgamma receptor and complement-dependent effector mechanisms. J Virol 85: 11567–11580. 21917960 10.1128/JVI.05859-11 PMC3209272

[21] Binley J (2009) Specificities of broadly neutralizing anti-HIV-1 sera. Curr Opin HIV AIDS 4: 364–372. 20048699 10.1097/COH.0b013e32832e06fe

[22] Li Y, Svehla K, Louder MK, Wycuff D, Phogat S, et al (2009) Analysis of neutralization specificities in polyclonal sera derived from human immunodeficiency virus type 1-infected individuals. J Virol 83: 1045–1059. 19004942 10.1128/JVI.01992-08 PMC2612402

[23] Pinna D, Corti D, Jarrossay D, Sallusto F, Lanzavecchia A (2009) Clonal dissection of the human memory B-cell repertoire following infection and vaccination. Eur J Immunol 39: 1260–1270. 19404981 10.1002/eji.200839129 PMC3864550

[24] Han T, Marasco WA (2011) Structural basis of influenza virus neutralization. Ann N Y Acad Sci 1217: 178–190. 21251008 10.1111/j.1749-6632.2010.05829.x PMC3062959

[25] Karlsson Hedestam GB, Fouchier RA, Phogat S, Burton DR, Sodroski J, et al (2008) The challenges of eliciting neutralizing antibodies to HIV-1 and to influenza virus. Nat Rev Microbiol 6: 143–155. 18197170 10.1038/nrmicro1819

[26] Crill WD, Hughes HR, Delorey MJ, Chang GJ (2009) Humoral immune responses of dengue fever patients using epitope-specific serotype-2 virus-like particle antigens. PLoS One 4: e4991. 19337372 10.1371/journal.pone.0004991 PMC2659788

[27] Oliphant T, Nybakken GE, Austin SK, Xu Q, Bramson J, et al (2007) Induction of epitope-specific neutralizing antibodies against West Nile virus. J Virol 81: 11828–11839. 17715236 10.1128/JVI.00643-07 PMC2168772

[28] Lai CY, Tsai WY, Lin SR, Kao CL, Hu HP, et al (2008) Antibodies to envelope glycoprotein of dengue virus during the natural course of infection are predominantly cross-reactive and recognize epitopes containing highly conserved residues at the fusion loop of domain II. J Virol 82: 6631–6643. 18448542 10.1128/JVI.00316-08 PMC2447043

[29] Heinz FX (1986) Epitope mapping of flavivirus glycoproteins. Adv Virus Res 31: 103–168. 2428213 10.1016/s0065-3527(08)60263-8

[30] Heinz FX, Mandl C, Berger R, Tuma W, Kunz C (1984) Antibody-induced conformational changes result in enhanced avidity of antibodies to different antigenic sites on the tick-borne encephalitis virus glycoprotein. Virology 133: 25–34. 6199892 10.1016/0042-6822(84)90422-7

[31] Verrier F, Nadas A, Gorny MK, Zolla-Pazner S (2001) Additive effects characterize the interaction of antibodies involved in neutralization of the primary dualtropic human immunodeficiency virus type 1 isolate 89.6. J Virol 75: 9177–9186. 11533181 10.1128/JVI.75.19.9177-9186.2001 PMC114486

[32] Ndifon W, Wingreen NS, Levin SA (2009) Differential neutralization efficiency of hemagglutinin epitopes, antibody interference, and the design of influenza vaccines. Proc Natl Acad Sci U S A 106: 8701–8706. 19439657 10.1073/pnas.0903427106 PMC2688967

[33] Lok SM, Kostyuchenko V, Nybakken GE, Holdaway HA, Battisti AJ, et al (2008) Binding of a neutralizing antibody to dengue virus alters the arrangement of surface glycoproteins. Nat Struct Mol Biol 15: 312–317. 18264114 10.1038/nsmb.1382

[34] Dowd KA, Jost CA, Durbin AP, Whitehead SS, Pierson TC (2011) A dynamic landscape for antibody binding modulates antibody-mediated neutralization of West Nile virus. PLoS Pathog 7: e1002111. 21738473 10.1371/journal.ppat.1002111 PMC3128118

[35] Cockburn JJ, Navarro Sanchez ME, Fretes N, Urvoas A, Staropoli I, et al (2012) Mechanism of dengue virus broad cross-neutralization by a monoclonal antibody. Structure 20: 303–314. 22285214 10.1016/j.str.2012.01.001

[36] Pierson TC, Kuhn RJ (2012) Capturing a Virus while It Catches Its Breath. Structure 20: 200–202. 22325768 10.1016/j.str.2012.01.014 PMC3733089

[37] Midgley CM, Flanagan A, Tran HB, Dejnirattisai W, Chawansuntati K, et al (2012) Structural analysis of a dengue cross-reactive antibody complexed with envelope domain III reveals the molecular basis of cross-reactivity. J Immunol 188: 4971–4979. 22491255 10.4049/jimmunol.1200227 PMC3364712

[38] Austin SK, Dowd KA, Shrestha B, Nelson CA, Edeling MA, et al (2012) Structural basis of differential neutralization of DENV-1 genotypes by an antibody that recognizes a cryptic epitope. PLoS Pathog 8: e1002930. 23055922 10.1371/journal.ppat.1002930 PMC3464233

[39] Wahala WM, Donaldson EF, de Alwis R, Accavitti-Loper MA, Baric RS, et al (2010) Natural strain variation and antibody neutralization of dengue serotype 3 viruses. PLoS Pathog 6: e1000821. 20333252 10.1371/journal.ppat.1000821 PMC2841629

[40] Sukupolvi-Petty S, Austin SK, Engle M, Brien JD, Dowd KA, et al (2010) Structure and function analysis of therapeutic monoclonal antibodies against dengue virus type 2. J Virol 84: 9227–9239. 20592088 10.1128/JVI.01087-10 PMC2937608

[41] Brien JD, Austin SK, Sukupolvi-Petty S, O'Brien KM, Johnson S, et al (2010) Genotype-specific neutralization and protection by antibodies against dengue virus type 3. J Virol 84: 10630–10643. 20702644 10.1128/JVI.01190-10 PMC2950583

[42] Nelson S, Jost CA, Xu Q, Ess J, Martin JE, et al (2008) Maturation of West Nile virus modulates sensitivity to antibody-mediated neutralization. PLoS Pathog 4: e1000060. 18464894 10.1371/journal.ppat.1000060 PMC2330159

[43] Daffis S, Kontermann RE, Korimbocus J, Zeller H, Klenk HD, et al (2005) Antibody responses against wild-type yellow fever virus and the 17D vaccine strain: characterization with human monoclonal antibody fragments and neutralization escape variants. Virology 337: 262–272. 15919103 10.1016/j.virol.2005.04.031

[44] Kaufmann B, Vogt MR, Goudsmit J, Holdaway HA, Aksyuk AA, et al (2010) Neutralization of West Nile virus by cross-linking of its surface proteins with Fab fragments of the human monoclonal antibody CR4354. Proc Natl Acad Sci U S A 107: 18950–18955. 20956322 10.1073/pnas.1011036107 PMC2973864

[45] Throsby M, Geuijen C, Goudsmit J, Bakker AQ, Korimbocus J, et al (2006) Isolation and characterization of human monoclonal antibodies from individuals infected with West Nile Virus. J Virol 80: 6982–6992. 16809304 10.1128/JVI.00551-06 PMC1489037

[46] Vogt MR, Moesker B, Goudsmit J, Jongeneelen M, Austin SK, et al (2009) Human monoclonal antibodies against West Nile virus induced by natural infection neutralize at a postattachment step. J Virol 83: 6494–6507. 19386704 10.1128/JVI.00286-09 PMC2698525

[47] Goncalvez AP, Purcell RH, Lai CJ (2004) Epitope determinants of a chimpanzee Fab antibody that efficiently cross-neutralizes dengue type 1 and type 2 viruses map to inside and in close proximity to fusion loop of the dengue type 2 virus envelope glycoprotein. J Virol 78: 12919–12928. 15542644 10.1128/JVI.78.23.12919-12928.2004 PMC525008

[48] de Alwis R, Smith SA, Olivarez NP, Messer WB, Huynh JP, et al (2012) Identification of human neutralizing antibodies that bind to complex epitopes on dengue virions. Proc Natl Acad Sci U S A 109: 7439–7444. 22499787 10.1073/pnas.1200566109 PMC3358852

[49] Zlatkovic J, Stiasny K, Heinz FX (2011) Immunodominance and functional activities of antibody responses to inactivated West Nile virus and recombinant subunit vaccines in mice. J Virol 85: 1994–2003. 21147919 10.1128/JVI.01886-10 PMC3067796

[50] Wahala WM, Silva AM (2011) The human antibody response to dengue virus infection. Viruses 3: 2374–2395. 22355444 10.3390/v3122374 PMC3280510

[51] Wahala WM, Huang C, Butrapet S, White LJ, de Silva AM (2012) Recombinant dengue type 2 viruses with altered e protein domain III epitopes are efficiently neutralized by human immune sera. J Virol 86: 4019–4023. 22278250 10.1128/JVI.06871-11 PMC3302537

[52] Wahala WM, Kraus AA, Haymore LB, Accavitti-Loper MA, de Silva AM (2009) Dengue virus neutralization by human immune sera: role of envelope protein domain III-reactive antibody. Virology 392: 103–113. 19631955 10.1016/j.virol.2009.06.037 PMC2746956

[53] Pond WL, Ehrenkranz NJ, Danauskas JX, Carter MJ (1967) Heterotypic serologic responses after yellow fever vaccination; detection of persons with past St. Louis encephalitis or dengue. J Immunol 98: 673–682. 6022887

[54] Kayser M, Klein H, Paasch I, Pilaski J, Blenk H, et al (1985) Human antibody response to immunization with 17D yellow fever and inactivated TBE vaccine. J Med Virol 17: 35–45. 2995571 10.1002/jmv.1890170106

[55] Cherrier MV, Kaufmann B, Nybakken GE, Lok SM, Warren JT, et al (2009) Structural basis for the preferential recognition of immature flaviviruses by a fusion-loop antibody. EMBO J 28: 3269–3276. 19713934 10.1038/emboj.2009.245 PMC2771083

[56] Cardosa MJ, Wang SM, Sum MS, Tio PH (2002) Antibodies against prM protein distinguish between previous infection with dengue and Japanese encephalitis viruses. BMC Microbiol 2: 9. 12019028 10.1186/1471-2180-2-9 PMC113253

[57] de Alwis R, Beltramello M, Messer WB, Sukupolvi-Petty S, Wahala WM, et al (2011) In-depth analysis of the antibody response of individuals exposed to primary dengue virus infection. PLoS Negl Trop Dis 5: e1188. 21713020 10.1371/journal.pntd.0001188 PMC3119640

[58] Dormitzer PR, Grandi G, Rappuoli R (2012) Structural vaccinology starts to deliver. Nat Rev Microbiol 10: 807–813. 23154260 10.1038/nrmicro2893

[59] Heinz FX, Kunz C (1981) Homogeneity of the structural glycoprotein from European isolates of tick-borne encephalitis virus: comparison with other flaviviruses. J Gen Virol 57: 263–274. 6172553 10.1099/0022-1317-57-2-263

[60] Clarke DH, Casals J (1958) Techniques for hemagglutination and hemagglutination-inhibition with arthropod-borne viruses. Am J Trop Med Hyg 7: 561–573. 13571577 10.4269/ajtmh.1958.7.561

[61] Li L, Lok SM, Yu IM, Zhang Y, Kuhn RJ, et al (2008) The flavivirus precursor membrane-envelope protein complex: structure and maturation. Science 319: 1830–1834. 18369147 10.1126/science.1153263

