# Supplementary figures and images for: Dissection of Antibody Specificities Induced by Yellow Fever Vaccination
Source: PLoS Pathog. 2013 Jun 20;9(6):e1003458. doi: 10.1371/journal.ppat.1003458 (PMC3688551; doi:10.1371/journal.ppat.1003458)

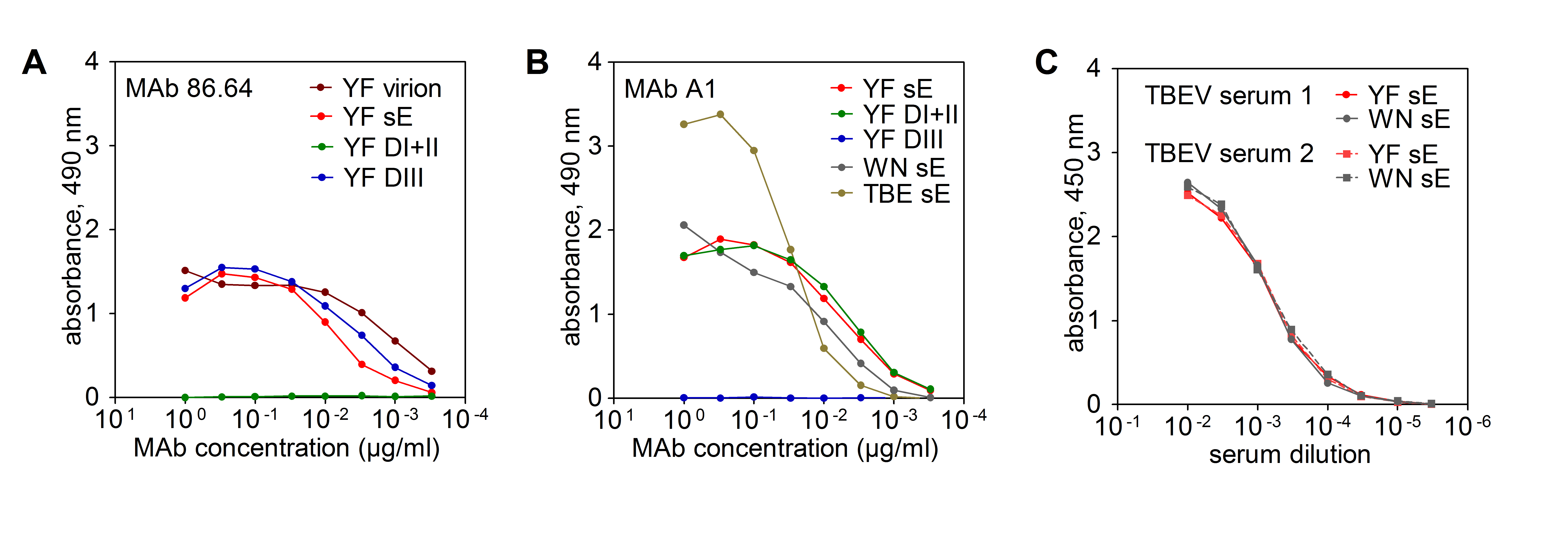

Supplement: Figure S2 — Sensitivities and specificities of YF virion and recombinant antigen ELISAs for the detection of YF virus-specific antibodies. (A) Reactivity with the DIII-specific MAb 86.64. (B) Reactivity with the cross-reactive fusion peptide loop-specific MAb A1. (C) Reactivity with TBE virus post-vaccination sera. (TIF) [file ppat.1003458.s002.tif]

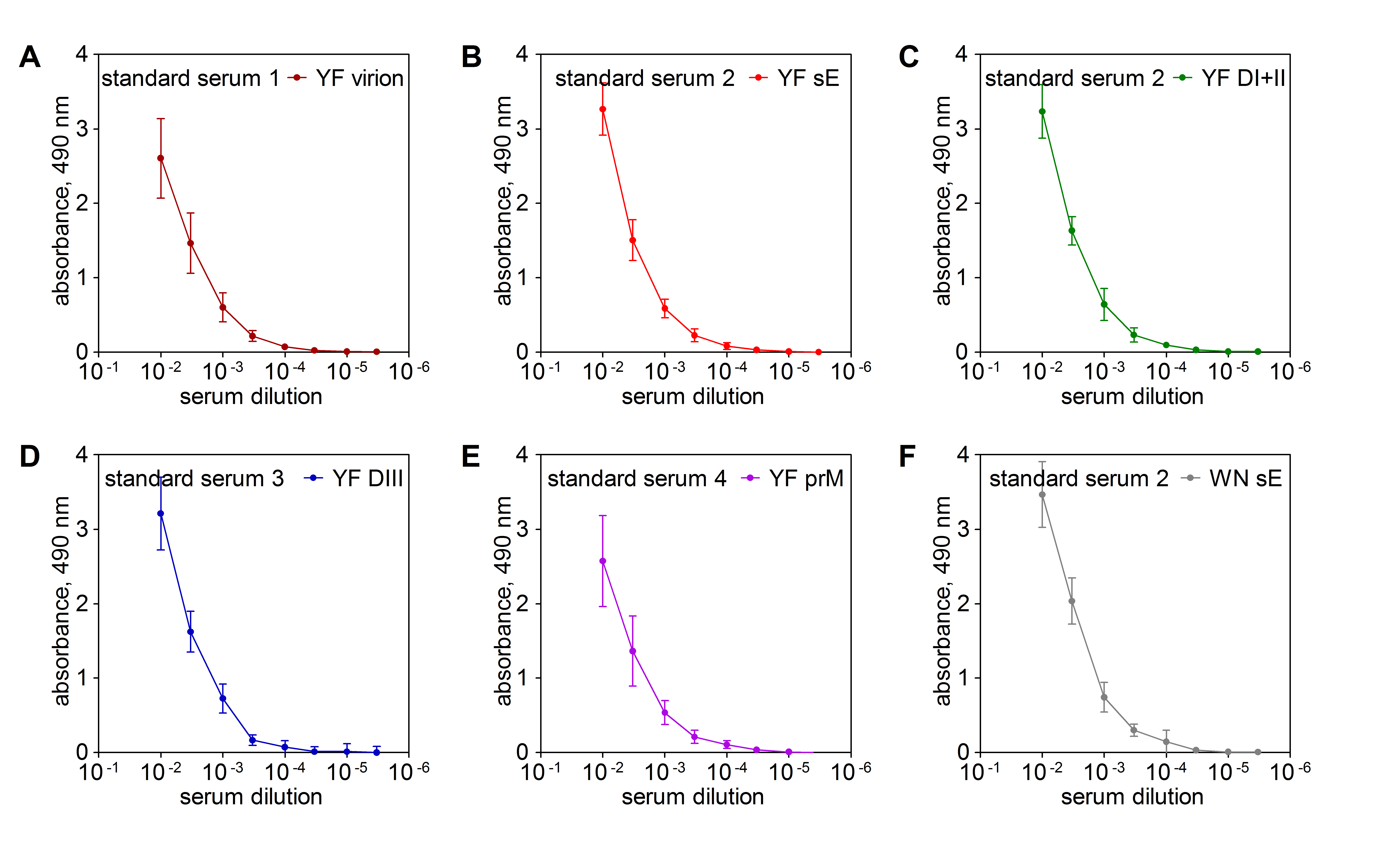

Supplement: Figure S3 — ELISA reactivities of standard sera with YF virion (A), YF sE (B), YF DI+II (C), YF DIII (D), YF prM (E), and WN sE (F). Error bars represent standard deviations, which were calculated from at least three independent experiments. (TIF) [file ppat.1003458.s003.tif]
