# Supplementary material for: Dissection of Antibody Specificities Induced by Yellow Fever Vaccination
Source: PLoS Pathog. 2013 Jun 20;9(6):e1003458. doi: 10.1371/journal.ppat.1003458 (PMC3688551; doi:10.1371/journal.ppat.1003458)
Supplement: Text S1 — describes the characterization of recombinant proteins used in the study and the standardization of ELISAs with these antigens. (DOCX) [file ppat.1003458.s005.docx]

**Supporting information Text S1:**

Characterization of recombinant proteins: The recombinant proteins used in this study were analyzed for purity, oligomeric structure, glycosylation, and the presence of disulfide bonds as shown in Figure S1. The heterogeneous migration of strep-tag containing proteins in SDS-PAGE (Figure S1A) is caused by the double strep-tag and not seen after its enzymatic removal by enterokinase (data not shown). The oligomeric structure of YF sE was assessed by SDS-PAGE analysis after chemical cross-linking in comparison to TBE sE (Figure S1B) and by sucrose gradient sedimentation analysis of YF and TBE sE at pH 8.0 and 6.0. Acidic pH induces dissociation of dimeric sE into monomers [62] (Figure S1C). Both of these analyses indicated that YF sE-strep was a monomer, which is similar to the previously described recombinant sE of West Nile virus [63,64], but different from the dimeric TBE virus sE ([65], Figure S1BC). The presence of carbohydrate side chains was assessed for YF sE-strep, YF DI-II-strep and YF prM-strep using SDS-PAGE analysis after treatment with endoglycosidase PNGase F (Figure S1D). Protein glycosylation was verified for YF prM, whereas YF sE was shown to lack a carbohydrate side chain, consistent with its sequence [66]. The presence of disulfide bridges in the recombinant YF proteins was verified by Western blot analyses with monoclonal and polyclonal sera under reducing and non-reducing conditions, which in all cases revealed loss of reactivity after reduction (Figure 1SE).

Standardization of ELISAs with recombinant proteins: The sensitivities and specificities of the virion-, sE-, DI+II-, and DIII-ELISAs were assessed by comparative titrations of monoclonal antibodies and polyclonal sera (Figure S2 A-C). As can be seen in Figure S2A, the DIII-specific MAb 86.64 reacted similarly with all DIII-containing antigens, but not with the recombinant YF DI+II. As expected, the flavivirus cross-reactive MAb A1 (recognizing the fusion peptide loop in DII [18]) reacted with all of the DII-containing antigens but not with DIII (Figure S2B). The validity of YF and WN sEs for detecting broadly flavivirus cross-reactive antibodies was verified with a TBE virus post-vaccination serum (Figure S2C).

For the quantification of ELISA data, we used internal standard curves of positive sera from YF vaccinated individuals that were defined to contain 1000 arbitrary IgG units and run in parallel in all of the assays. In order to be used as standards in the assays, sera had to have high titers with the corresponding antigens and an appropriate curve-fitting pattern for accurate quantification of IgG content. No single serum sample displayed these properties with all recombinant antigens, therefore four different standard serum samples were used (Figure S3).

References:

62. Stiasny K, Allison SL, Marchler-Bauer A, Kunz C, Heinz FX (1996) Structural requirements for low-pH-induced rearrangements in the envelope glycoprotein of tick-borne encephalitis virus. J Virol 70: 8142-8147.

63. Kanai R, Kar K, Anthony K, Gould LH, Ledizet M, et al. (2006) Crystal structure of west nile virus envelope glycoprotein reveals viral surface epitopes. J Virol 80: 11000-11008.

64. Nybakken GE, Nelson CA, Chen BR, Diamond MS, Fremont DH (2006) Crystal structure of the West Nile virus envelope glycoprotein. J Virol 80: 11467-11474.

65. Rey FA, Heinz FX, Mandl C, Kunz C, Harrison SC (1995) The envelope glycoprotein from tick-borne encephalitis virus at 2 A resolution. Nature 375: 291-298.

66. Rice CM, Lenches EM, Eddy SR, Shin SJ, Sheets RL, et al. (1985) Nucleotide sequence of yellow fever virus: implications for flavivirus gene expression and evolution. Science 229: 726-733.
