# Supplementary material for: Dissection of Antibody Specificities Induced by Yellow Fever Vaccination
Source: PLoS Pathog. 2013 Jun 20;9(6):e1003458. doi: 10.1371/journal.ppat.1003458 (PMC3688551; doi:10.1371/journal.ppat.1003458)
Supplement: Text S2 — describes Materials and Methods pertaining to data shown in the supporting figures. (DOCX) [file ppat.1003458.s006.docx]

**Supporting methods Text S2:**

Chemical cross-linking with dimethylsuberimidate (DMS)

This analysis was carried out essentially as previously described [67]. In brief, 2 µg purified recombinant YF sE-strep and TBE sE-strep were incubated with 10mM DMS for 30 min at room temperature. The reaction was stopped by adding ethanolamine to a final concentration of 10 mM and incubating the mixture for an additional 15 min at room temperature. Proteins were precipitated with trichloroacetic acid (TCA) and analyzed by electrophoresis on a sodium dodecyl sulfate (SDS)–5% polyacrylamide gel using a continuous phosphate-buffered system [68] and protein bands were visualized with BioSafe Coomassie Stain (Bio-Rad)

Sedimentation analysis

Three micrograms purified recombinant YF sE-strep and TBE sE-strep were applied to 7-20% (wt/wt) continuous sucrose gradients in TAN buffer pH 8.0 (50 mM triethanolamine, 100 mM NaCl) or 2-(N-morpholino)ethanesulfonic acid buffer pH 6.0 (MES; 50 mM MES, 100 mM NaCl) containing 0.1% Triton X-100 [62,69]. Samples were centrifuged for 20 h at 38,000 rpm at 15°C in a Beckman SW40 rotor and fractionated by upward displacement. The presence of E protein in the fractions was determined by ELISA.

PNGaseF treatment

Two micrograms purified recombinant YF sE-strep, YF DI+II-strep, and YF prM-strep were incubated with 60 units of PNGase F (New England Biolabs) according to the manufacturer’s protocol. PNGaseF-treated proteins were precipitated with TCA and analyzed by 12% SDS-polyacrylamide gel electrophoresis (PAGE) according to Laemmli. Protein bands were visualized with BioSafe Coomassie Stain (Bio-Rad).

Western blot

One hundred nanograms of recombinant protein – with and without prior reduction - were subjected to 12% SDS-PAGE according to Laemmli, blotted onto polyvinylidene difluoride (PVDF) membranes (Bio-Rad) with a Bio-Rad Trans-Blot semidry transfer cell, and detected and visualized immunoenzymatically as previously described [70].

YF virus-specific monoclonal and polyclonal antibodies

The YF virus-specific monoclonal antibodies (MAbs) 86.64, 86.25, and 86.13 were obtained from the European Virus Archive (http://www.european-virus-archive.com/). Hybridoma cells were grown in RPMI 1640 medium (Sigma) containing 10% fetal calf serum (FCS), penicillin and streptomycin. MAbs were purified by protein A Sepharose High Performance (GE Healthcare Life Sciences) according to the manufacturer's recommendations. The rabbit polyclonal serum was generated by immunization of rabbits with the YF vaccine.

ELISA with YF virus specific monoclonal antibodies

Microtiter plates were coated overnight at 4°C with pre-determined optimized dilutions of purified recombinant antigens or virus in carbonate buffer, pH 9.6. Plates were blocked with PBS pH 7.4 containing 2% lamb serum for 20 min at 37°C. Three-fold serial dilutions (starting at 1µg/ml) of MAbs were then added for 1 h at 37°C. Bound antibodies were detected using peroxidase-labeled rabbit anti-mouse IgG (Pierce) as previously described [18].

References:

67. Allison SL, Schalich J, Stiasny K, Mandl CW, Kunz C, et al. (1995) Oligomeric rearrangement of tick-borne encephalitis virus envelope proteins induced by an acidic pH. J Virol 69: 695-700.

68. Maizel JV, Jr. (1971) Polyacrylamide gel elctrophoresis of viral proteins. Methdos Virol 5: 179-246.

69. Stiasny K, Allison SL, Schalich J, Heinz FX (2002) Membrane interactions of the tick-borne encephalitis virus fusion protein E at low pH. J Virol 76: 3784-3790.

70. Schalich J, Allison SL, Stiasny K, Mandl CW, Kunz C, et al. (1996) Recombinant subviral particles from tick-borne encephalitis virus are fusogenic and provide a model system for studying flavivirus envelope glycoprotein functions. J Virol 70: 4549-4557.
